# Supplementary material for: External Validation of a Radiomics Model for the Prediction of Complete Response to Neoadjuvant Chemoradiotherapy in Rectal Cancer
Source: Cancers (Basel). 2022 Feb 21;14(4):1079. doi: 10.3390/cancers14041079 (PMC8870201; doi:10.3390/cancers14041079)
Supplement: Supplementary file 1 [file cancers-14-01079-s001.zip › cancers-1551701-supplementary.pdf]

Supplementary Materials

Table S1 : MRIs Acquisition parameters

Table S2 : Composition of each model and importance of each feature

Figure S1 : Example of manually tumor segmentation

Table S1. MRIs Acquisition parameters.

Aquisition parameters	Siemens 1.5T (institution 1)	Philips 1.5T (institution 1)	Siemens 1.5T (institution 1)	Siemens 1.5T (institution 2)	Philips Achieva 3T (institution 2)	Siemens 1.5T (institution 2)	Siemens 3T (institution 2)
Number of patients	20	6	38	25	13	4	18
Magnetic field strength (Tesla)	1.5	1.5	1.5	1.5	3	1.5	3
T2-weighted							
Matrix (pixels)	320 x 320	400 x 55	320 x 320	320 x 320	300 x 256	256 x 256	320 x 320
Field of view (mm)	300 x 300	220 x 220	220 x 220	200 x 200	240 x 240	200 x 200	200 x 200
ET (ms)	120	100	107	90	90	77	90
RT (ms)	5800	10400	6800	5500	4000	4900	5600
Slice thickness (mm)	3	3	3.5	4	3	3.5	3
Diffusion							
Matrix (pixels)	128 x 108	116 x 118	126 x 126	128 x 160	116 x 116	154 x 103	160 x 120
Fiels of view (mm)	249 x 249	240 x 240	249 x 249	200 x 200	240 x 240	249 x 249	240 x 240
ET (ms)	75	80	88	72	74	83	60
RT (ms)	7000	2500	3500	4200	4800	4700	4200

Slice thickness (mm)	4	3	5	4	4	4	4
Gradient	B50-400-1000	B1000	B50-800-1000	B50-800	B0-1000	B50-800	B0-400-800

Abbreviations: T: Tesla, mm: milimeters, ms: milisecond, ET: Echo-Time, RT: Repetition Time

Table S2. Composition of each model and importance of each feature.

Model	Feature	Importance (%)
Clinical	Tumour grade	9.5
	Tumour stage	90.5
Radiomic	HGLRE_align_Diffusion	1.9
	Entropy_Hist_T2	14.1
	Elongation_T2	21.4
	Elongation_Diffusion	26.3
	Energy_Hist_T2	36.3
Combined	HGLRE_align_Diffusion	0.7
	Tumour grade	2.6
	Elongation_T2	6.7
	Elongation_Diffusion	11.2
	Entropy_Hist_T2	12.3
	Energy_Hist_T2	19.6
	Tumour stage	47.0
Combat_Radiomic	Elongation_Diffusion	0.2
	Mean_Hist_T2	1.0
	GLNU_norm_align_T2	5.4
	StandardDeviation_T2	7.6
	Energy_Hist_T2	11.0
	HGLZE_Diffusion	16.1
	Variance_Hist_T2	58.7
Combat_Combined	Mean_Hist_T2	1.1
	GLNU_norm_align_T2	1.6
	Energy_Hist_T2	4.2

	Elongation_Diffusion	7.0
	HGLZE_Diffusion	7.9
	StandardDeviation_T2	10.5
	Tumour stage	27.5
	Variance_Hist_T2	40.3

Abbreviations: HGLRE: High Gray Level Run Emphasis, T2: T2-weighted sequence, Hist: Histogram, GLNU: Grey-Level Non-Uniformity, HGLZE: High Gray Level Zone Emphasis

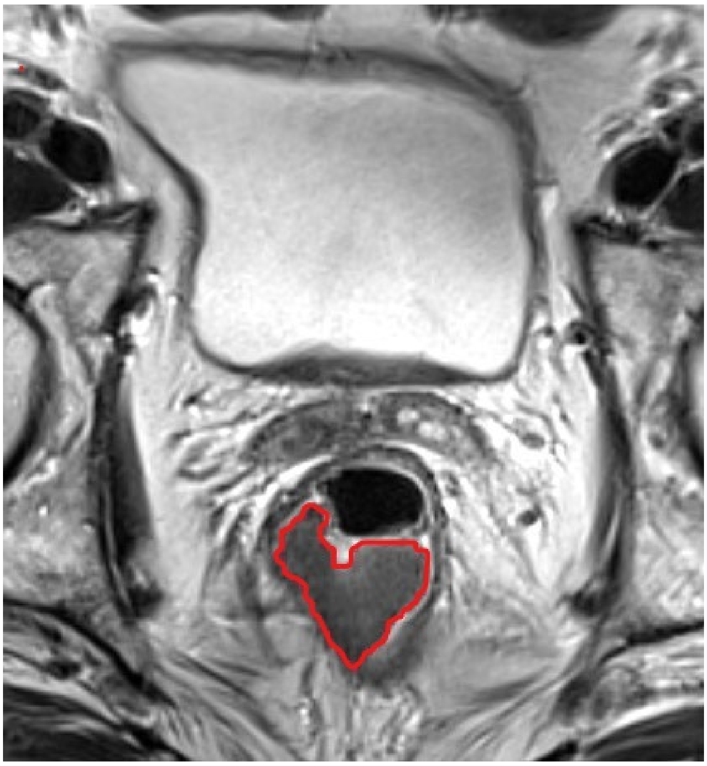

Figure S1 : Example of manually tumor segmentation.
